# Observations on the Case of Mollities Ossium Described in the Preceding Article; with Some General Remarks on That Disease

**Published:** 1787

**Authors:** John Hunter

**Affiliations:** Surgeon Extraordinary to the King


VIII. *Obſervations on the Caſe of Mollities Oſſium deſcribed in the preceding Article; with ſome general Remarks on that Diſeaſe. Communicated in a Letter to Dr.* Simmons *by* John Hunter, *Eſq. F. R. S., Surgeon Extraordinary to the King.*

I BEG leave to return you my thanks for your attention in ſending me the very curious arm of the ſubject affected by the mollities oſſium; and as you propoſe to publiſh the caſe in the next part of your Medical Journal, I have ſent you ſome general obſervations upon the diſeaſe, with a few remarks on the diſſection tion of this arm: thefe, if you think they will render the account more complete, may be annexed to it.

This difeafe, commonly known by the term Mollities Offium, in the adult, is, in my opinion, a fpecies of the rickets which is pecular to youth, and arifes from a difpofition for abforption of the fubftance of a bone, or a difproportion between the powers of depofiting new matter and thofe of removing the old: this, in many inftances, has been carried to a much greater extent in the full grown than in the young fubject; for in the moft rickety child I have ever feen there was always fome earth in the bones; but I have feen them in the adult fo foft from the lofs of the calcareous earth, that they have been almoft as flexible as a tendon, and fuch bones have had little or nothing of the appearance of the natural animal part of a bone when only deprived of the earth; therefore they are not compofed of the original animal part, but a new depofit of animal fubftance in a different form.

In fome of thefe bones it is curious to fee the effects produced by the two different difpofitions. In one part of the bone the offific difpofition is taking place, and forming bone in the the cavity, and in ſome places on the ſurface, of the orignal bone ; but the diſpoſition for abſorption goes on too faſt for the oſſific, and even abſorbs portions of the newly-ſet-up oſſifications.

Previous to my examination of the arm from the perſon whoſe caſe has been communicated to you, I injected the arteries, with a view to ſee if any alteration had taken place in that ſyſtem of veſſels; and in the diſſection I obſerved the following appearances :

The muſcles, blood veſſels, nerves, and abſorbents, as far as they could be examined, were in no way remarkable.

The os humeri was more vaſcular than is common, from which we may conclude the other ſyſtems of veſſels were alſo increaſed ; and it is probable that the abſorbents were principally ſo, for we may remark, that whenever a part has greater actions to carry on than what are natural to it, the number of veſſels which are the active parts of the body are always increaſed.

The bones of the fingers were lighter and leſs compact than common. Thoſe of the metacarpus were in ſome degree ſofter ; the radius and ulna were ſtill more ſo, and the os humeri was, if the expreſſion is admiſſible, completely diſeaſed.

As I had not an opportunity of examining the different bones of the body, nothing can be aſcertained reſpecting the diſeaſe being confined to particular bones, or its affecting equally thoſe of the trunk and extremities; but the ribs could not have been equally diſeaſed with the os humeri, without affecting the reſpiration ſo materially as to have made the patient very uncomfortable from that cauſe, which, as appears in the account, was not the caſe; for although the diaphragm might have acted very well, it is neceſſary that it ſhould have a circle of fixed points to act from to produce its effects in reſpiration.

The os humeri retained its ſhape externally, and the cartilages at both the articulations appeared not in the leaſt affected.

The component parts of the bone were totally altered, the ſtructure being very different from other bones, and wholly compoſed of a new ſubſtance, reſembling a ſpecies of fatty tumour, giving the appearance of a ſpongy bone deprived of its earth and ſoaked in ſoft fat. This ſtructure was moſt remarkable under the external lamella, which was not ſo much al-

 tered, tered, making a kind of cafe for the other, and having the periofteum adhering to it, the whole could be readily cut with a knife.

Near to the condyles a portion of this fubftance had been deficient for nearly two inches of the bone's length, and the outer fhell at this part filled with a bloody fluid contained in cells. This part of the bone readily bent, and in the living body had been miftaken for a fracture; there was a fimilar appearance a little higher up than the middle of the bone for nearly an inch in length.

The radius and ulna exhibited the fame ftructure and appearances as the os humeri, and were alfo free from any abfolute fracture, but had portions of the internal ftructure deficient, and the fpace filled up by a bloody fluid.

It is probable that thofe parts which gave way firft to the action of the mufcles and other circumftances, and which appeared to be fractures, had thofe parts afterwards abforbed from a kind of neceffity, ftimulating the abforbents to remove the parts fo affected.

*Leicefter Square,*

March 1, 1787.

